# A comparative analysis to forecast apartment burglaries in Vienna, Austria, based on repeat and near repeat victimization

**DOI:** 10.1186/s40163-018-0083-7

**Published:** 2018-08-20

**Authors:** Philip Glasner, Shane D. Johnson, Michael Leitner

**Affiliations:** 1 Department of Geoinformatics–Z_GIS, University of Salzburg, Salzburg, Austria; 2 SynerGIS Informationssysteme GmbH, Vienna, Austria; 3 Jill Dando Institute of Security and Crime Science, University College London, London, UK; 4 Department of Geography and Anthropology, Louisiana State University, Baton Rouge, LA USA

**Keywords:** Repeats, Near repeats, Burglary, Predictive mapping, Crime prevention, Vienna

## Abstract

In this paper, we introduce two methods to forecast apartment burglaries that are based on repeat and near repeat victimization. While the first approach, the “heuristic method” generates buffer areas around each new apartment burglary, the second approach concentrates on forecasting near repeat chain links. These near repeat chain links are events that follow a near repeat pair of an originating and (near) repeat event that is close in space and in time. We name this approach the “near repeat chain method”. This research analyzes apartment burglaries from November 2013 to November 2016 in Vienna, Austria. The overall research goal is to investigate whether the near repeat chain method shows better prediction efficiencies (using a capture rate and the prediction accuracy index) while producing fewer prediction areas. Results show that the near repeat chain method proves to be the more efficient compared to the heuristic method for all bandwidth combinations analyzed in this research.

## Introduction

Early scientific studies showed that crime events are spatially concentrated (Guerry 1833; Quetelet 1835). In recent years, an increasing number of studies have revealed that crime events are not only spatially but also temporally clustered (e.g., Polvi et al. 1991; Sagovsky and Johnson 2007). The fact that crime events are spatially and temporally concentrated, leads to pattern analysis that can be, and has been, used in crime prevention practice. Simply put, areas of clustered crime events are worthy of crime prevention attention because research suggests (e.g., Johnson and Bowers 2004a; Townsley et al. 2003) that it is very likely that other crime events will occur quickly nearby. Studies have shown that resource allocation to risky locations reduces crime, without displacing offending to areas nearby (e.g., Bowers et al. 2011; Braga et al. 2014).

While high-risk locations may be predicted with some accuracy, it may be inefficient to *permanently* deploy police resources to these locations. For example, research suggests that (some) crime clusters are unstable and may move between different locations (e.g., Johnson and Bowers 2004b). Moreover, in the case of police patrols, there may be only marginal gains of deterring disorderly and criminal behavior to be made by increasing the amount of time that officers spend at particular locations (Koper 1995). For such reasons, the optimal deployment of resources may involve a more dynamic allocation strategy, whereby resources are only deployed for short amounts of time before being moved to other locations (Koper 1995; Telep et al. 2014).

Due to the ever-changing nature of crime event clusters, several different analytical methods and tools have been developed and evaluated to forecast future crime locations. Among these analytical methods are popular and commonly used approaches like kernel density estimation, risk-terrain modelling, and methods that rely on principles of repeat and near repeat victimization. Kernel density estimation is used to generate risk surfaces to show where crime clustered historically (e.g., Johnson et al. 2008; Leitner et al. 2018). Based on these so-called retrospective clusters, actions on crime prevention, such as resource allocation, can be planned. However, kernel density estimation only considers the crime location, and ignores the temporal component. Bowers et al. (2004) suggested a weighted kernel density estimation with recent crime events receiving a higher weight in the calculation. More recently, Rosser et al. (2017) extended this approach by performing predictions at the street segment level.

For a long-term perspective, risk terrain modeling (Caplan et al. 2011) shows promising results to combat crime. This method does not focus on individual crime events and their distributions, but estimates the influence of environmental factors on crime (for a European example, see Kocher and Leitner 2015). Criminogenic factors such as bars, metro stations, or parks are weighted and layered together to produce an overall risk surface. As these risk maps rarely change, this approach is more useful for long-term crime prevention activities. Returning to short-term forecasts, the principle of repeat and near repeat victimization has motivated several recent approaches to crime forecasting. Repeat victimization occurs when a person or property is victimized multiple times, whereas “near repeats” are said to have occurred when offenses occur at locations near to those recently victimized (Morgan 2001). Studies, including interviews with offenders, show that once a crime occurs, it is likely that the same offender will return to repeat crimes at or close to the initial target (Ericsson 1995; Pease 1998; Weisel 2005; Summers et al. 2010; Johnson et al. 2009). It also shows that repeat victimization, when it occurs, tends to happen shortly after an initial crime event (e.g., Polvi et al. 1991), but that the risk of repeat victimization decreases in the weeks after the initial event (Ratcliffe 2009). Such regularities have informed a number of predictive approaches which assume that crime is more likely at or nearby victimized locations in the near future (for a more detailed discussion of predictive methods, see Groff and La Vigne 2002; Bowers and Johnson 2014). A further suggestion that has been proposed but not tested (Caplan et al. 2013) is to combine hotspot mapping, near repeat analysis, and risk terrain modeling.

In this paper, we introduce and compare two approaches for forecasting apartment burglary^1^ locations that are both based on principles of repeat and near repeat victimization. The first is a simple heuristic approach, while the second uses the concept of “near repeat chains” (Johnson et al. 2007a; Townsley 2007). Traditional near repeat analyses (for an exception, see Johnson and Bowers 2004b) consider only pairs of events (e.g. an initial crime and a subsequent near repeat), ignoring the fact that a series of near repeats might follow an initial event. The analysis of near repeat chains addresses this.

The paper is organized as follows: The next section provides a review of the literature, focusing on repeat and near repeat victimization in particular. Research questions and objectives are then discussed, followed by a discussion of forecasting techniques and an analysis of apartment burglaries for the city of Vienna, Austria. The only previous geospatial predictive research concerned with crime data from Vienna was conducted by Glasner and Leitner (2017), who analyzed the impact that the weekday has on near repeat victimization of street robberies. To the best of our knowledge, no one has previously analyzed repeat and near repeat patterns of apartment burglaries in Vienna, let alone in Austria. This paper provides new insights into forecasting methods based on repeats and near repeats. Additionally, the paper reflects on the influence of spatial and temporal parameter settings used in near repeat analysis and how this information could be useful for crime prevention strategies of law enforcement agencies.

## Theoretical background

Crime prevention actions can be particularly effective when applied to those who are most at risk of victimization, such as households or individuals (Weisel 2005). Several interventions intended to reduce repeat victimization have been evaluated with impressive results. The Kirkholt Burglary Prevention Project implemented in Rochdale, UK (Forrester et al. 1988), was one of the first such crime prevention initiatives. This project focused on reducing (high levels of repeat) residential burglaries using crime prevention interventions, such as improved property security. The evaluation of the project suggested that the approach employed led to an 80% reduction in repeat victimization and an overall reduction in burglary of 53% in the project area (Forrester et al. 1988).

As discussed above, research suggests that those near to previously victimized homes are also at an elevated risk shortly after a crime occurs. Besides residential burglaries (e.g., Bowers and Johnson 2004; Johnson and Bowers 2004a), significant near repeat patterns have been identified for crimes to include shootings (Ratcliffe and Rengert 2008), street robberies (e.g., Haberman and Ratcliffe 2012; Glasner and Leitner 2017), gun assaults (Wells et al. 2012), and insurgent activities (Townsley et al. 2008; Braithwaite and Johnson 2015).

To date, two theories have been proposed to explain why repeats and near repeats occur. According to the “flag” account (e.g. Pease 1998) it is assumed that the risk of victimization is time-stable but unevenly distributed. The idea, in the context of residential burglary, is that there are some general characteristics associated with properties that attract (different) offenders and, therefore, that homes that possess these characteristics will be repeatedly victimized. Such characteristics may include, but are not limited to, low security, minimal natural surveillance, or good escape routes. In the case of this account, repeat victimizations are assumed to be independent and hence committed by different offenders. Such an assumption is at odds with empirical research (Everson and Pease 2001; Bernasco 2008; Johnson et al. 2009) that clearly shows that most repeats and near repeats are the work of returning offenders. Moreover, the theory would suggest that there should be little to no pattern in the timing of (near) repeat victimizations (but see, Johnson 2008). As discussed, there is a clear time course to (near) repeat victimization, questioning the extent to which this account can fully explain observed patterns.

On the other hand, the “boost” account and “optimal foraging” theory are more likely to explain why repeats and near repeats occur. The “boost” account is referred to as a heightened, “boosted” risk following an initial offense (Pease 1998). A (serial) offender decides to return to a well-known property, location, or area in his/her awareness space, where he/she uses prior gained knowledge to reoffend. In the case of a burglary, for example, offenders know the property and what they have left behind. This type of search behavior has been described as an optimal foraging strategy (similar to those observed across species) by Bowers and Johnson (2004). The offender as a forager tries to maximize the benefits of their criminal activity within some constraints. These constraints include experience and knowledge of opportunities, as well as the travel time and effort involved in committing crimes. Crimes are therefore committed at locations where the offender maximizes perceived benefits and minimizes the expected risks. Once an area has been exhausted by the best burglary opportunities, the offender moves on to a new location (Johnson et al. 2009).

In line with this, near repeat burglaries have been found to be more likely to be committed by the same offender than non-near repeat burglaries (Bernasco 2008; Johnson et al. 2009). Put differently, serial offenders often commit sequences of crimes at the same or neighboring properties shortly after one another. Following an initial event, there may be a series of (near) repeats, which form what Townsley (2007) and Johnson et al. (2007b) refer to “near repeat chains” (for a detailed treatment, see Davies and Marchione 2015). Wells et al. (2012) refer to these as “near repeat sets”, Behlendorf et al. (2012) describe these related events as “microcycles”, while Santos and Santos (2015a) introduce them as “micro-time hot spots”. Haberman and Ratcliffe (2012) suggest that the disruption of multiple-event chains with police resource allocation would be a useful crime prevention strategy. In their analysis of armed street robberies in Philadelphia, PA, USA, Haberman and Ratcliffe (2012) found out that there is a 30% chance that a near repeat pair will have another near repeat within 7 days and 1200 feet (approx. 366 m), making it a “strong finding with real proactive potential”.

## Approach

### Research objectives

The research objectives in this paper are to test and evaluate two different forecasting methods applied to apartment burglaries, both of which are based on the principles of repeat and near repeat victimization. The first approach involves the calculation of buffer areas (of different sizes) around each apartment burglary that occurs. Based on the “boost” account, it is assumed that offenders will return to the same and nearby locations of these incidents. These forecasted areas are valid for just a few days, because the risk of another event taking place is assumed to be elevated close to a previous event but for a limited period of time. The prediction accuracy of this method is first evaluated by comparing it to expectation, assuming that there is no pattern to the timing and location apartment burglaries. This first approach is similar to the method carried out by Fielding and Jones (2012) in their study in Greater Manchester. In that study, the authors created a 400 m buffer around domestic burglaries, while coloring the buffers to reflect changes in the risk based on the time elapsed since the burglary occurred.

Results from the first method are compared to a second approach, which is motivated by the concept of near repeat chains. This second approach was tested as the first created many prediction areas, due to there being a large number of apartment burglaries that occurred in Vienna each day. To narrow down the number of prediction areas, the second technique relies on the assumption that following a first near repeat, subsequent near repeats are more likely to occur. For this method, buffer areas are hence derived for every near repeat (as opposed to every event). An important research question is whether the near repeat chain technique results in similar or better prediction accuracies than the first approach. A second research question analyzes whether the prediction accuracy of each techniques differs from chance expectation. Although we know that crime is not randomly distributed, the comparison with chance expectation is a fundamental baseline of most statistical approaches. We know that crime is not random nor should comparison prediction areas be random. However, police resource allocation in Austria is somewhat arbitrary and this approach is still common practice. The approach of randomly placing prediction areas is designed to approximate arbitrary resource allocation. It is hypothesized that non-random prediction areas show better prediction accuracies. To the best of our knowledge, an extensive analysis of the predictive accuracy of an approach based on near repeat chains and their potential to forecast future residential burglaries has not been researched before.

The remainder of the paper is organized as follows: “Approach” section concludes with a short discussion about data preparation, which is followed by a “Methodology” section. The subsequent “Results” section presents and discusses results of the comparative analysis to forecast apartment burglaries in Vienna, Austria, based on repeat and near repeat victimization. The final two sections are “Discussion” and “Conclusion”.

### Data preparation

Vienna is home to approx. 1.85 million people, which is about one-fifth of all residents in Austria. Austria records approximately 550,000 reported crime incidents per year, of which 38% (210,000) occur in Vienna (Criminal Intelligence Service Austria 2014). Of those, roughly 6000 apartment burglaries are reported every year. Although the volume of burglary incidents in Austria has been decreasing since 2004 (Grafl et al. 2012), apartment burglaries in Vienna are a significant problem compared to other Austrian, respectively European, cities. The Criminal Intelligence Service Austria does not analyze residential burglaries together, but differentiates between residential burglaries to apartments and residential burglaries to houses. In this study, only apartment burglaries are analyzed. All crime data reported in Austria are centrally stored in a large database, the so-called Security Monitor. The database is administered by the Criminal Intelligence Service Austria and was made available to the authors for the purposes of this study.

Apartment burglary incidents that occurred in apartments in Vienna over a 3-year period from November 2013 to October 2016 are analyzed in this research. During this 3-year period, there was a total of 17,649 burglaries, with an average of 13.4 apartment burglaries per day. Each recorded crime includes the XY coordinate of the incident location. The positional accuracy of the geocoded data is very high for most crime types, with the exception of larceny or pickpocketing (Glasner and Leitner 2017). Of the total number of incidents, 99.8% (17,611 events) were geocoded to a unique address of an apartment building, and only 0.2% (38 events) are referenced to the street segment. These 38 events are included in this study.

As victims may not be home during an offense, the date and time of burglaries are recorded as the earliest and latest date/time they could have occurred. As described by Townsley et al. (2000), research on repeat and near repeat victimization is only reliable if locations are recorded accurately, and when the time window between start and end date of the crime event is not too large. Therefore, only apartment burglaries that occurred within a time window of 72 h were used in this research. People who work in Vienna but live in the countryside often leave their apartments on Friday and return on either Sunday or Monday—an average interval of 72 h. As a consequence of applying this inclusion criteria, 17.6% (3098 events) of all apartment burglaries were excluded. This means that 14,551 apartment burglary events, in total, were included in the subsequent analysis. For apartment burglary events with different start and end dates, the end date of an offense was consistently used in what follows.

### Methodology

#### Assessing the repeat and near repeat pattern

As a preliminary exercise, the first step in the analyses reported here involved establishing whether a pattern of repeat and near repeat victimization existed in the data. This analysis was completed using the Near Repeat Calculator (NRC) developed by Ratcliffe (2009), which implements the method described in Johnson et al. (2007a). Briefly, each crime event is compared to every other and the distance and time between them calculated. Ultimately, the aim of the test is to determine whether there are observed more event pairs that occur close in space and time than would be expected if the timing and location of events were independent. The latter is estimated using a Monte Carlo simulation for which the observed data are permuted by shuffling the dates on which the burglaries took place (this produces a distribution for which there can be no relationship between the timing and location of events). This is repeated many times to enable estimation of the likelihood that the observed distribution could have occurred on a chance basis. A statistical significance of p = 0.05 is considered an appropriate minimum threshold within the social sciences and can be achieved with 20 iterations of a Monte Carlo simulation. Here, we use 99 iterations, which enables the estimation of p-values as low as 0.01.

The NRC requires the user to select the spatial and temporal bandwidths used for analysis. The spatial bandwidth is a threshold distance used to define which events are considered to have occurred close to a previous event. The temporal bandwidth is the time span between sequential events. In previous research, many different spatial and temporal parameters have been used to analyze near repeat patterns. Johnson et al. (2007a) reviewed near repeat patterns of residential burglary in ten different areas using spatial bandwidths between 200 and 1200 m and a temporal time span between 2 and 8 weeks. The choice of parameters is something of a limitation, because analysts can set parameters arbitrarily. In this study, the average length of a street segment in Vienna is used as the spatial bandwidth. The calculated average length of all street segments is roughly 90 m but for simplicity, 100 m are used and multiplied by factors of 3, 5, 7, and 9 to include longer bandwidths. These five spatial (100, 300, 500, 700, and 900 m) and five temporal parameter bandwidths (1, 3, 5, 7, and 9 days) are used and evaluated in the framework of a sensitivity analysis. Results should provide recommendations to the police on which parameters to use in practice. However, such recommendations are context specific and may only be applied to the current and similar study areas.

#### Forecast methods

**Fig. 1:**
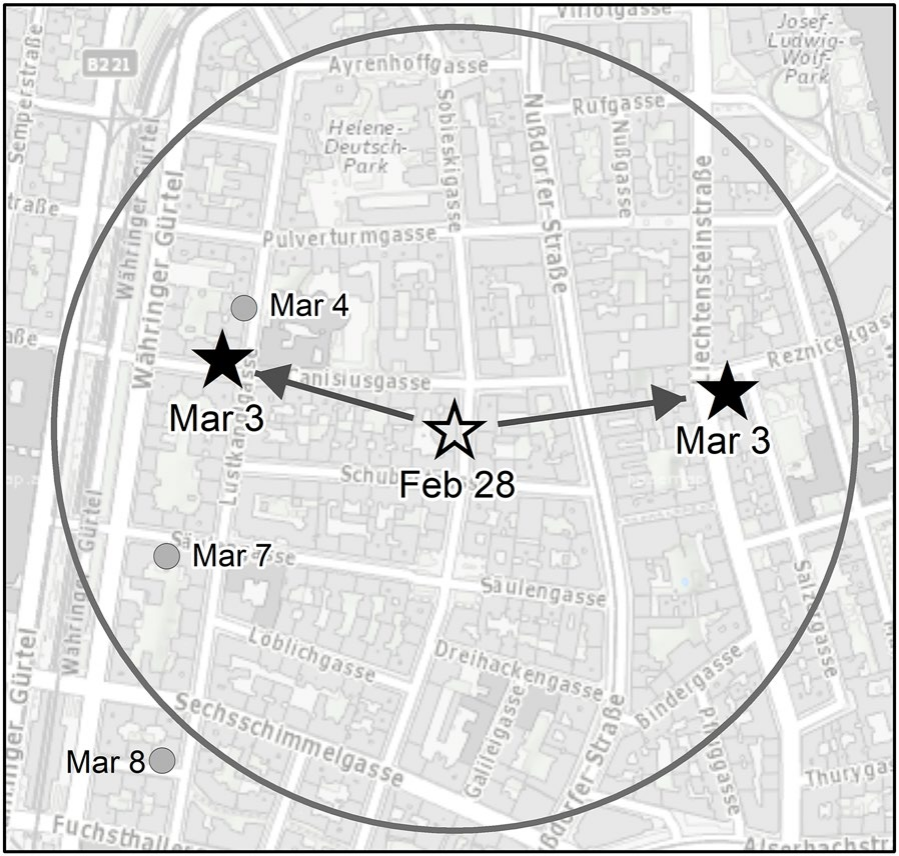
The principle of the heuristic method: buffer areas are generated around each event that occurred on a specific day (Feb 28), searching for near repeat apartment burglaries that occur close in space (300 m) and time (3 days)

As discussed, the research focuses on the evaluation of two methods for forecasting future crime events. The first involves creating geographical buffer areas around each incident for a given time frame. For example, for today’s forecast of future burglary events, dissolved buffer areas of (say) 300 m radius were generated around all burglaries that occurred on the previous (say) 3 days (Fig. 1). The buffer areas are used to generate prediction areas within which the risk of another burglary is expected to be temporarily increased. These areas are valid for a few days, for example, 3 days, representing the number of days which forecasts last when making predictions. This time span is referred to as the forecast period. In this study, we name this approach the “heuristic method” because the method is simple to use and easy to apply. A conceptually similar approach, as discussed by Perry et al. (2013), appears to work best for burglaries, because of this crime type’s strong near repeat effects.

**Fig. 2:**
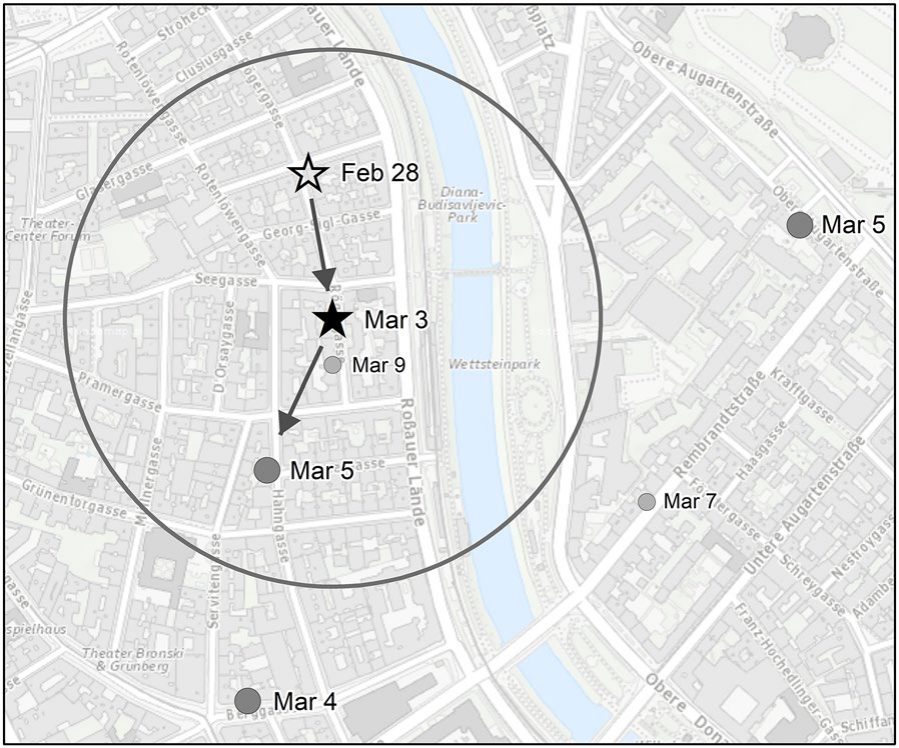
The principle of the near repeat chain method: A near repeat pair creates a prediction area and searches for following near repeat chain links in close space (300 m) and time (3 days)

The second method takes a slightly different approach for which the number of prediction areas produced per day is reduced compared to the first method. For example, if 15 new apartment burglaries occur every day, 15 new prediction areas need to be generated using the heuristic method. If the forecasting period is 5 days long, then about 75 predictions are generated. In fact, it is challenging to develop preventive strategies for so many prediction areas. Therefore, another approach is introduced that is based on near repeat chains. This approach identifies near repeat pairs that are both close in space and time. Dissolved buffer areas are then created around the near repeat events (the second event in the event pair), so that these near repeats act as originating events for subsequent near repeats. Hereafter, we refer to this approach as the “near repeat chain method”. Figure 2 shows an initiating apartment burglary (Feb 28) with a near repeat (Mar 3) close in both space (300 m) and time (3 days). Based on this near repeat pair, a prediction area is drawn as a buffer of the near repeat event. In this prospective analysis, the method searches for further (near) repeats that are close in space and time (see event on Mar 5 within the circle).

To evaluate the predictive accuracy of both approaches, a comparison is made to a random distribution of prediction areas which are generated in the following way. Prediction areas are sampled at random using data about the unique locations of all 14,551 apartment burglaries located in the study area. In each case, the same amount of prediction areas that were generated using either the heuristic or the near repeat chain method are selected at random from all historic apartment burglary locations (from November 2013 to October 2016) across Vienna. This represents a simple Poisson process and doing this once provides an estimate of the expected accuracy, assuming that prediction accuracy reflects nothing more than a random process. However, one sample may be misleading and hence a Monte Carlo simulation is used to repeat the process 99 times, which allows us to test the likelihood of observing a particular result with a level of statistical significance equal to 0.01. This level of statistical significance would suggest that the pattern observed would be expected to occur by chance only once in one hundred times (Besag and Diggle 1977).

In the real-world, forecast areas would likely be used for a specified amount of time to direct crime prevention activities. This time is referred to as the forecast period throughout the remainder of this article. In this prospective analysis, five different forecast periods are evaluated, namely 1, 3, 5, 7, and 9 days. In addition, five different spatial bandwidths (100, 300, 500, 700, and 900 m) are used. These spatial and temporal parameters represent various bandwidths that are within the range of bandwidths used in earlier studies (e.g., Bowers and Johnson 2004; Johnson et al. 2007a; Haberman and Ratcliffe 2012). This study discusses the impact of these bandwidths on predicting apartment burglaries in Vienna. In total, this results in 125 parameter combinations that are evaluated for their predictive power and predictive accuracy.

#### Assessing predictive power and predictive accuracy

To assess predictive accuracy, the results for both methods are first compared using the capture rate, and second, using the prediction accuracy index. The benefit of using the two methods in tandem is that the prediction accuracy index (PAI) speaks to efficiency—how many offenses are captured per percentage of study area, whereas the capture rate tells what fraction of offenses might be prevented.

The capture rate represents the proportion of predicted events from the total number of events in the dataset. The larger the proportion, the larger the possible reduction in events due to possible crime prevention strategies employed by the police. Because this rate does not take the size of prediction areas into account, the PAI is additionally calculated to evaluate predictive accuracy. First introduced by Chainey et al. (2008), this index measures the proportion of predicted events from the total number of events (capture rate) divided by the proportion of the size of the prediction area(s) related to the entire study area. The larger the PAI, the more events are predicted and/or the smaller the size of the prediction areas is. This means that the larger the PAI, the more accurately forecasts can be made. As an alternative to the PAI, the search efficiency rate (SER) (Bowers and Johnson 2004) could have been used, as well. We are aware that both the PAI and SER (as well as other prediction measures that take a prediction area into account) are problematic in this case, since the entire space of prediction areas include places where apartment burglaries could not actually happen. Unfortunately, building footprints or the actual number of apartments were unknown and therefore were not available as a more suitable denominator.

All calculations were automatically processed using a Python script using the ArcPy site-package of Esri’s ArcGIS. For each of the spatial and temporal parameter combinations, the script calculated the number and sizes of the prediction areas generated per day, and analyzed which “future” events occurred within prediction areas, flagging these events as predicted events. Based on these results, the two evaluation indices (capture rate and PAI) were calculated. Examining the findings for the different methods for different spatial and temporal bandwidths enables an assessment of the sensitivity of the approach to different parameter combinations and provides an assessment of the optimal parameter settings.

## Results

### Assessing space–time clustering using the Near Repeat Calculator (NRC)

In this section, we investigate whether there is a pattern of repeat and/or near repeat victimization for apartment burglaries in Vienna. Given observed patterns elsewhere, it is expected that apartment burglaries will cluster in space and in time. Table 1 shows the results from the NRC. By default, spatial distances start from the same location, where a previous apartment burglary has already occurred, followed by, for example, 1–300 m, 301–600 m, and 601–900 m. Apartment burglaries that occur at the same location as a previous event represent repeat victimizations. In Table 1, however, spatial distances start from the same location and continue with spatial distances from 1 to either 100, 300, 500, 700, and 900 m (near repeat victimization). The temporal distance starts from 0 days, which enumerates apartment burglaries that occur on the same day as a previous apartment burglary at the same location. By default, temporal distances start from 0 to, for example, 3 days, followed by 4–6 days, and 7–9 days. In Table 1, each category starts from 0 days to 1, 3, 5, 7, and 9 days respectively. In fact, the values in Table 1 are a compilation of 25 results from the NRC. The values in Table 1 can be interpreted as providing an indication of the risk that future apartment burglaries will happen at the same location as a previous crime, and/or at distances from 1 to 100, 300, 500, 700, and 900 m, and from 0 to 1, 3, 5, 7, and 9 days (near repeat victimization). More precisely, each value indicates the ratio of the number of observed pairs that occur within a particular distance and time as each other over the average number expected. The larger the value, the larger the difference between the observed count of pairs and that expected (calculated assuming no space–time pattern exists). The significance level is based on Monte Carlo simulations and indicates whether the risk level is statistically significant or not.

**Table 1:** Repeat and near repeat risk values (observed over mean expected frequencies) of apartment burglaries in Vienna, Austria from November 2013 to November 2016

Spatial distance	Temporal distance				
	0–1 day	0–3 days	0–5 days	0–7 days	0–9 days
Same location	98.84	22.52	13.48	9.88	8.02
1–100 m	2.98	1.75	1.52	1.39	1.38
1–300 m	1.96	1.35	1.23	1.17	1.15
1–500 m	1.60	1.23	1.17	1.12	1.12
1–700 m	1.45	1.20	1.13	1.10	1.09
1–900 m	1.34	1.15	1.10	1.08	1.07

All values are statistically significant (*p* = 0.01)

The value in upper-left of Table 1 of 98.84 indicates that almost 100 times as many event pairs occur within 0–1 days of each other at the same location than would be expected, assuming that the timing and location of burglaries were independent. It suggests that there is a high risk of repeat victimization within 1 day after a burglary occurs at an apartment. It appears that the ratio of observed to expected event pairs decays over space and time. For the highest spatial and temporal bandwidth combination (1–900 m and 0–9 days) considered, the ratio is only marginally greater than one indicating only a subtle difference between the numbers of pairs (of events that occur at these distances and times from each other) observed and those expected. Similar observations have been found in many other studies (e.g., Bowers et al. 2004). Overall, it can be concluded that a significant near repeat pattern exists for apartment burglaries in Vienna for the 3-year period considered.

### Predicting apartment burglaries using the heuristic method

This section focuses on the accuracy of the repeat and near repeat forecasting approach that uses the heuristic method. Essentially, for each apartment burglary in the dataset, the routine written searches for subsequent burglaries that occur within a given space and time of the burgled home. As discussed, different parameter values were used to define what is considered close in space (100, 300, 500, 700, and 900 m), and time (1, 3, 5, 7, and 9 days). This routine is repeated for each day for which data exists and flags apartment burglaries that are identified as “near repeats”. Of course, a single event cannot be predicted more than once and it should be noted that—as with previous studies of this kind—predictions are produced for the day after the interval used to generate predictions.

Table 2 shows evaluation results in the form of capture rates for the heuristic method. Recall that the capture rate measures the proportion of predicted events from the total number of events in the dataset. The values in Table 2 thus represent the percentage of apartment burglaries that are near repeats or near repeat chain links of the total number of apartment burglaries in the dataset (i.e., 14,551 burglaries). Overall, capture rates range from 1.5 to 80.5%. This means that if the police would have successfully prevented every single near repeat apartment burglary, the number of apartment burglaries would have potentially decreased by as much as 80.5%. A potentially preventable proportion of 80.5% of apartment burglaries is certainly stunning, however, this is more a theoretical than practical value. The main reason is that increasing spatial bandwidths leads to a non-linear increase in the size of prediction areas. This is also combined with longer forecasting periods which represent the number of days that a forecast lasts. This, in turn, leads to a higher number of apartment burglaries that are identified within these increasingly larger areas. Large prediction areas in total, resulting from an average of 13.4 new individual prediction areas per day (14,551 apartment burglaries divided by 1086 analysis days) are hardly suitable for crime prevention strategies.

**Table 2:** Capture rates of apartment burglaries using the heuristic method (in %) in Vienna, Austria from November 2013 to November 2016

Spatial distance (m)	Forecast period				
	1–1 day	1–3 days	1–5 days	1–7 days	1–9 days
0–100	1.5**	3.5**	5.2**	6.8**	8.4**
0–300	5.9**	14.7**	21.6**	27.8*	33.1
0–500	12.5**	29.1**	40.3	50.0	57.0
0–700	20.3**	42.4**	55.7	65.3	71.6
0–900	27.9**	54.1**	67.3	75.6	80.5

Statistical significances are obtained by Monte Carlo simulations

* Capture rates possess a level of significance of p < 0.05

** Capture rates possess a level of significance of p = 0.01

However, it should be noted that the number of prediction areas alone can be misleading, since prediction areas can overlap and because they depend on the spatial bandwidth selected. For this reason, we examine the size of prediction areas explicitly. Table 3 shows the mean sizes of prediction areas per day (whether or not they overlap) in hectares (1 ha = 10,000 m^2^). As a rule of thumb, 1 ha is often referred to as the size of one soccer field. This means that on average, prediction areas generated using a 100 m bandwidths and a 1 day forecasting period (upper-left entry in Table 3) cover an area approximately equal to 38 soccer fields each day.

**Table 3:** Total sizes of prediction areas per day for apartment burglaries in Vienna from November 2013 to November 2016 using the heuristic method (in hectares)

Spatial distance (m)	Forecast period				
	1–1 day	1–3 days	1–5 days	1–7 days	1–9 days
0–100	38	113	189	265	340
0–300	331	992	1653	2315	2976
0–500	882	2645	4408	6172	7935
0–700	1645	4934	8223	11,513	14,802
0–900	2572	7715	12,858	18,001	23,145

The advantage of the PAI as a prediction evaluation method is that it takes the size of the prediction area vis-a-vis the entire study area into account. It calculates the proportion of predicted apartment burglaries by the total number of apartment burglaries divided by the proportion of the size of prediction areas by the entire study area. As such, the PAI can be used to compare across different crime types or, as is the case of this study, across different parameter combinations.

Table 4 lists PAI results for the heuristic method. Recall that the larger the PAI, the more accurate predictions are. Results suggest the most efficient predictions are obtained for smaller spatial and temporal bandwidths. Increasing the spatial bandwidths has a greater influence on PAI values, than does increasing the temporal bandwidths. Both bandwidths show a distinctive decline in PAI values of up to 900 m and up to 9 days. However, the PAI decreases more rapidly with increased spatial distance than with increased temporal bandwidth.

**Table 4:** PAI of near repeats of apartment burglaries using the heuristic method for Vienna from November 2013 to November 2016

Spatial distance (m)	Forecast period				
	1–1 day	1–3 days	1–5 days	1–7 days	1–9 days
0–100	16.1**	11.9**	10.8**	10.7**	10.2**
0–300	6.8**	6.4**	5.9**	5.7*	5.5
0–500	5.7**	5.2**	4.7	4.6	4.5
0–700	5.0**	4.4**	4.0	3.8	3.7
0–900	4.5**	3.9**	3.5	3.4	3.2

Statistical significances are obtained by Monte Carlo simulations

* PAIs possess a level of significance of p < 0.05

** PAIs possess a level of significance of p = 0.01

There is no commonly used combined method to evaluate the predictive power (capture rate) and the predictive accuracy (PAI). In this research, both evaluation metrics can be combined by using threshold values. For example, with a capture rate of at least 10%, meaning that at least 10% of apartment burglaries can be predicted, which of the spatial and temporal parameter combinations yields the largest PAI value? To give an example, the combination of up to 300 m spatial bandwidth combined with a forecasting period of up to 3 days shows the highest PAI value of 6.4 (Table 4). Using this parameter combination, a capture rate of 14.7% for apartment burglaries is calculated (Table 2). For the same parameter combination, the total size of prediction areas amount to 992 ha (Table 3). Given that a prediction area with a radius of 300 m is approximately 28 ha large, this means that for such a parameter combination, there would be about 35 prediction areas per day (992 ha divided by 28 ha), over which crime prevention activity would need to be targeted.

The approach of comparing observed patterns of prediction areas to randomized prediction areas and their potential to predict apartment burglaries reveals significant differences, when using different spatial and temporal bandwidths. The shorter the spatial and temporal bandwidths are, the more statistically significant are both evaluation indices for the observed compared to the random distribution of apartment burglaries. In contrast, no statistical significance in the prediction accuracy can be observed, when using the heuristic method with larger spatial and temporal bandwidths. In Tables 2 and 4 (as well as in Tables 5 and 7) evaluation values received one asterisk, when the analysis of the observed apartment burglaries showed a statistically significant pattern (p < 0.05). Values with two asterisks are statistically significant at p = 0.01. In general, it can be concluded that when using the heuristic method to forecasting apartment burglaries, selected spatial and temporal bandwidths work best for shorter spatial and temporal bandwidths.

**Table 5:** Capture rates of apartment burglaries using the near repeat chain method (in %) in Vienna, Austria from November 2013 to November 2016

Spatial distance (m)	Temporal distance/forecast period				
	Up to 1 day	Up to 3 days	Up to 5 days	Up to 7 days	Up to 9 days
0–100	0.3**	0.6**	0.9**	1.4**	1.9**
0–300	1.3**	4.6**	8.5**	12.9**	17.3**
0–500	4.0**	14.5**	24.5**	35.1**	43.2*
0–700	9.5**	27.4**	42.4**	54.2*	62.6*
0–900	16.0**	40.9**	57.3**	68.0*	74.3*

Temporal distances range from 0 to *n* days; forecasting periods range from 1 to *n* days. Statistical significances estimated using a Monte Carlo simulation

* Capture rates possess a level of significance of p < 0.05

** Capture rates possess a level of significance of p = 0.01

### Predicting apartment burglaries using the near repeat chain method

The near repeat chain method is designed to reduce the number of prediction areas, by preselecting areas that are already prone to near repeats. As discussed, rather than assuming near repeats will following every burglary, the near repeat chain method identifies existing repeat and near repeat pairs and assumes that a future (near) repeat is more likely near to one or both of these events (see Davies and Marchione 2015). It is based on the assumption that repeat offenders adopt foraging strategies by exploiting areas that are (currently) familiar to them for a short period of time. Moreover, that the occurrence of pairs of events that occur close in space and time likely signal the activity of such offenders and suggest that subsequent events are likely in the near future nearby.

The near repeat chain method was applied to the same crime data as the heuristic method in the previous section. The same spatial and temporal bandwidth combinations discussed in “Predicting apartment burglaries using the heuristic method” section were applied, and the findings compared and assessed using the same evaluation measures. Results of the capture rate are shown in Table 5 and the PAI in Table 7. Table 6 shows the sizes of prediction areas generated using the near repeat chain method. For reasons of clarity and comprehensibility, Tables 5, 6, 7 show results for which the number of days between the originating and the repeat event is the same as the forecasting period.

**Table 6:** Total sizes of prediction areas per day for apartment burglaries in Vienna from November 2013 to November 2016 using the near repeat method (in hectares)

Spatial distance (m)	Temporal distance and forecast period				
	Up to 1 day	Up to 3 days	Up to 5 days	Up to 7 days	Up to 9 days
0–100	3	12	23	36	51
0–300	51	232	489	813	1194
0–500	215	1021	2122	3476	4951
0–700	554	2530	5124	8079	11,179
0–900	1099	4776	9318	14,228	19,225

Temporal distances range from 0 to *n* days; forecasting periods range from 1 to *n* days

**Table 7:** PAI of near repeats of apartment burglaries using the near repeat method for Vienna from November 2013 to November 2016

Spatial distance (m)	Temporal distance and forecast period				
	Up to 1 day	Up to 3 days	Up to 5 days	Up to 7 days	Up to 9 days
0–100	23.4**	13.1**	11.7**	11.9**	11.3**
0–300	7.6**	6.9**	6.7**	6.4**	6.2**
0–500	6.2**	5.9**	5.4**	5.1**	4.8*
0–700	6.1**	5.0**	4.5**	4.2*	3.9*
0–900	5.7**	4.5**	3.9**	3.6*	3.3*

Temporal distances range from 0 to *n* days; forecast periods range from 1 to *n* days. Statistical significances are obtained by Monte Carlo simulations

* PAIs possess a level of significance of p < 0.05

** PAIs possess a level of significance of p = 0.01

Table 5 shows the capture rates, which can be interpreted as the predictable and potentially preventable proportion of all apartment burglaries. Capture rates increase with the spatial and temporal bandwidths and range from 0.3 to 74.3%. Compared to the respective results of the heuristic method in Table 2, it is evident that this method predicts fewer apartment burglaries. This is because there are fewer prediction areas which results in a smaller prediction areas (Table 6). However, the larger the spatial and temporal bandwidths, the smaller the difference in the capture rates is between the two methods.

Considering the prediction area sizes, Table 6 indicates that these range from 3 to 19,225 ha, which are smaller than for the heuristic method. This number takes overlapping prediction areas into account. A parameter combination of 0–300 m and a near repeat distance of 3 days results in an average prediction area of 232 ha/day. This compares to 992 ha of prediction areas per day that resulted from the same bandwidth combination using the heuristic method (compare Table 3). When comparing capture rates for both forecasting methods, the heuristic method clearly outperforms the near repeat chain method for this bandwidth combination, with a rate of 14.7% compared to 4.6%, respectively (compare Tables 2 and 5). In order for the near repeat chain method to achieve a similar capture rate of about 15%, the spatial bandwidth for the near repeat chain method would need to be increased to 500 m (to predict 14.5% of all apartment burglaries). With these capture rates for both evaluation methods, crime prevention strategies need to be planned for 992 ha for the heuristic method, and for 1021 ha for the near repeat chain method, respectively. In this example, the total prediction area for the heuristic method includes approx. 35 individual areas, while for the near repeat chain method it includes only 13 areas, respectively. While each of the 13 prediction areas are larger than each of the 35 areas, crime prevention strategies can focus on a fewer number of prediction areas of apartment burglaries, when applying the near repeat chain method.

Results for the PAI are shown in Table 7. PAI values range from 3.3 to 23.4. The larger the PAI, the more accurate the predictions are. As before, PAIs can be combined with capture rates from Table 5. For example, if a capture rate of at least 10% is required, the largest PAI with a value of 6.4 is calculated with a spatial bandwidth of 300 m, and with both a temporal bandwidth and forecasting period of 7 days.

Relative to the heuristic method, the PAI values are larger for the near repeat chain method for equivalent spatial and temporal bandwidth combinations (compare Tables 4 and 7). This means that the proportion of predicted apartment burglaries by prediction area is larger when focusing on predicting near repeat chain links. Using the above example, a bandwidth combination of 300 m and 3 days, results in a slightly higher PAI for the near repeat chain method, compared to the heuristic approach (6.9 compared to 6.4, respectively). Finally, both capture rates (Table 5) and PAI values (Table 7) are, without a single exception, statistically significant, at least, at p < 0.05.

## Discussion

The research investigated in this article falls under an often-discussed theme entitled “criminal predictive analytics”. The main research objective was to analyze and evaluate existing and newly developed methods to forecast the location and time of future apartment burglaries in the city of Vienna, Austria. All proposed forecasting methods were based on the repeat and near repeat victimization concept and are simple to implement, relative to some of the more complicated approaches discussed in the literature (e.g., Johnson et al. 2007b; Rosser et al. 2017; Mohler et al. 2011). A sensitivity analysis using several spatial and temporal bandwidth parameters for the (near) repeat approach was applied to evaluate the proposed forecasting methods. To the best knowledge of the authors, this is the first time that such predictive analytics concepts have been tested and evaluated with crime data in Austria.

As expected, this research showed that repeats and near repeats play an important role in apartment burglaries in Vienna, Austria. Using a dataset for a period of approximately 3 years (November 2013 to November 2016), statistically significant repeat and near repeat patterns were identified. This research revealed that a high proportion of all apartment burglaries in Vienna can be attributed to repeat and near repeat apartment burglary victimizations. General results from this research match findings from comparable international studies (e.g., Johnson et al. 2007b).

Two new and simple crime prediction methods were designed, tested, and evaluated in this article. The first method is a simple heuristic approach that places a buffer area of a user-selected radius around each new apartment burglary. This so-called prediction area is applicable for a short time period, referred to as the forecast period. As there are on average 13.4 new apartment burglaries per day in Vienna, this approach creates around 13.4 new prediction areas per day. This means that for a forecast period of 3 days, there would be, on average, about 40 prediction areas for the entire forecast period, or 67 prediction areas for a forecast period of 5 days.

The second method uses identified near repeat chains to predict future crime locations. Relative to the heuristic approach, this method generates smaller prediction areas. It has the advantage that the focus is on those areas that are already affected by near repeats. As suggested by Haberman and Ratcliffe (2012), near repeat chain links provide a “real proactive potential” to forecast, where crimes are more likely to occur. Davies and Marchione (2015) discuss the possibility to predict crime based on groups of events, rather than individual events. To our best knowledge, however, this is the first method that uses near repeat chains to forecast any type of crime event.

The analysis between the heuristic and the near repeat chain methods revealed stark differences in the number and sizes of prediction areas calculated by both forecasting methods. For same bandwidth combinations, the heuristic method always creates a higher number and larger sizes of prediction areas, compared to the near repeat chain method. This impacts the calculation of the capture rate for both forecasting methods, with the heuristic method clearly outperforming the near repeat chain method regarding this first evaluation measure. On the other hand, the near repeat chain method generates a smaller number and hence more manageable number of prediction areas, enabling crime prevention strategies to be more focused on the most critical areas of Vienna.

Results of this research show that both methods are suitable for predicting where and when future crimes are likely to occur. Using the PAI, both methods and different user-defined spatial and temporal bandwidth combinations can be compared and evaluated. The evaluation of five spatial and five temporal bandwidths reveal that the most accurate forecasts (highest PAI values) are obtained with the shortest bandwidth combinations for both the heuristic and the near repeat chain method. However, additional analysis shows that using the shortest bandwidth for both prediction measures would have decreased the potential prevention (capture rate) of predicted apartment burglaries by the least amount. Unfortunately, no measure currently exists that takes both predictive accuracy, as calculated with the PAI, and predictive power (potential prevention of predicted crimes as calculated with the capture rate) into account. However, this research proposes a possible solution to this shortcoming, by defining an optimal spatial and temporal threshold value for calculating the capture rate. After some testing, a spatial bandwidth threshold of 300 m and a temporal bandwidth threshold (both for finding near repeat pairs and for the forecast period) of 3 days was found to be most appropriate. Capture rates for this optimal bandwidth combination resulted in a value of 14.7% for the heuristic method and 4.6% for the near repeat chain method, respectively.

Overall, the near repeat chain method proves to be the more efficient of the two evaluation methods, since it resulted in higher PAI values than the heuristic method for all bandwidth combinations analyzed here. While for the heuristic method only the originating event is needed to make a forecast, a near repeat is additionally required to make a forecast using the near repeat chain method.

## Conclusion

The results of this research suggest that there is considerable potential for law enforcement in the city of Vienna to employ the concepts of repeat and near repeat victimization to forecast future apartment burglaries. The approaches discussed in this study can easily be interpreted by analysts and by police officers. Using automated workflows, the calculation of the results from these predictive analytic methods only takes a few minutes per day. While these methods work well for apartment burglaries, it does not necessarily mean that the principle of repeat and near repeat victimization is also suitable for other types of crime in the selected study area. The predictive methods investigated in this research may also only be applicable to the city of Vienna and may not show similar results in other cities around the world. Future research might thus test these methods for other types of crime and for other cities.

The analysis of repeat and near repeat victimization presents many opportunities for future research. As an alternative and a possible enhancement of the proposed predictive methods could be to do the analysis based on administrative boundaries, such as districts or police beats, in order to assess which level of aggregation or size of enumeration unit is more suitable in predicting near repeats. First tests at the district level show that districts that are closer to the city center experiencing higher risks and therefore more suitable for predicting apartment burglaries than districts further away. But underlying enumeration units do not necessarily have to be administrative units. It is also imaginable to define specific areas of interest, such as spatial and temporal hotspots of apartment burglaries and focus the analysis on areas where crime is clustered in both space and time. In addition, an alternative selection for a study area could be to use high-risk areas identified by the risk terrain modeling approach.

This research (and studies like it) does not include information on serial offenders. If this information were available, then a possible enhancement of the near repeat chain method would be to define prediction areas by serial offenders that committed crimes at both the originating and near repeat locations. Of course, such information is unlikely to be available at the present time given the time it takes to catch offenders and universally low rates of detection. However, prediction areas might only be created if both crime events of the near repeat pair share the same method of entry into an apartment (see Bowers and Johnson 2004).

Of course, the effectiveness of predictive policing relies not only on efficient forecasting methods, but also effective crime prevention strategies. Santos and Santos (2015a) suggest that effective responses implemented as soon as the near repeat chain begins will shorten its duration and severity. Results showed that tactical police interventions in Port St. Lucie, FL, USA, to near repeat chains led to significant decreases of 20% in thefts from vehicles (Santos and Santos 2015a) and residential burglary (Santos and Santos 2015b). However, research needs to continue to focus not only on where to deploy resources but what to do at such locations and how this might vary by context (e.g. Johnson et al. 2015).

## Abbreviations

NRC: Near Repeat Calculator
PAI: prediction accuracy index
SER: search efficiency rate

## Footnotes

1 Burglary to single- or multi-family houses or commercial burglary are not considered.
